# Protective Behaviors Associated With Gender During the 2018-2020 Ebola Outbreak in Eastern Democratic Republic of the Congo

**DOI:** 10.1001/jamanetworkopen.2021.47462

**Published:** 2022-02-16

**Authors:** Phuong N. Pham, Manasi Sharma, Kennedy Kihangi Bindu, Pacifique Zikomangane, Rachel C. Nethery, Eric Nilles, Patrick Vinck

**Affiliations:** 1 Harvard T.H. Chan School of Public Health, Boston, Massachusetts; 2 Harvard Medical School, Boston, Massachusetts; 3 University Libre des Pays des Grands Lacs, Quartier Himbi, Goma, République Démocratique du Congo

## Abstract

**Importance:**

In 2018 to 2020, the Democratic Republic of the Congo experienced the world’s second largest Ebola virus disease (EVD) outbreak, killing 2290 individuals; women were disproportionately infected (57% of all cases) despite no evidence of differential biological EVD risk. Understanding how gender norms may influence exposure to EVD, intensity, and prognosis as well as personal protective behaviors against the virus is important to disease risk reduction and control interventions.

**Objective:**

To assess whether men and women differ in personal protective behaviors (vaccine acceptance, health-seeking behaviors, physical distancing) and the mediating role of EVD information and knowledge, perceived disease risk, and social relations.

**Design, Setting, and Participants:**

This cross-sectional, multistage cluster survey study of 1395 randomly selected adults was conducted in the Ebola-affected regions of North Kivu from April 20, 2019, to May 10, 2019. Path analyses were conducted using structural equation modeling to examine associations among study variables. Statistical analysis was conducted from August 2019 to May 2020.

**Main Outcomes and Measures:**

The main behavioral outcomes of interest were (1) vaccine acceptance, (2) formal health care seeking, and (3) self-protective behaviors. The primary factor of interest was self-reported gender identity. We also assessed sociodemographic factors.

**Results:**

Among the study’s 1395 participants, 1286 (93%) had Nande ethnicity and 698 (50%) were women; the mean (SD) age was 34.5 (13.1) years. Compared with female participants, male participants reported significantly higher levels of education, wealth, and mobile phone access. There were associations found between gender and all EVD preventive behavioral outcomes, with evidence for mediation through EVD knowledge and belief in rumors. Men reported greater EVD knowledge accuracy compared with women (mean [SE] score for men: 12.06 [0.13] vs women: 11.08 [0.16]; *P* < .001), and greater knowledge accuracy was associated with increases in vaccine acceptance (β = 0.37; *P* < .001), formal care seeking (β = 0.39; *P* < .001), and self-protective behaviors (β = 0.35; *P* < .001). Lower belief in rumors was associated with greater vaccine acceptance (β = −0.30; *P* < .001), and greater EVD information awareness was associated with increased adoption of self-protective behaviors (β = 0.23; *P* < .001).

**Conclusions and Relevance:**

This survey study found gender differences in adopting preventive protective behaviors against EVD. These findings suggest that it is critical to design gender-sensitive communication and vaccination strategies, while engaging women and their community as a whole in any response to infectious disease outbreaks. Research on the potential link between gender and sociodemographics factors associated with disease risk and outcomes is needed.

## Key Points

**Question:**

Are gender differences in Ebola virus disease (EVD)–related information, knowledge, and perceptions associated with differences in protective behaviors?

**Findings:**

This survey study of 1395 randomly selected adults in Ebola-affected areas of eastern Democratic Republic of the Congo found that significant gender differences in preventive behavioral outcomes were mediated by gender differences in EVD knowledge and belief in rumors.

**Meaning:**

This study’s findings suggest that addressing gender differences in access to preventive and protective measures of information, communication messaging, and community engagement could enhance the effectiveness of outbreak control interventions.

## Teaser

This survey study investigates whether men and women differed in personal protective behaviors (vaccine acceptance, health-seeking behaviors, physical distancing) during the Eastern Democratic Republic of the Congo Ebola outbreak and assesses the mediating role of Ebola virus disease information and knowledge, perceived disease risk, and social relations.

## Introduction

From August 2018 to June 25, 2020, the world’s second largest Ebola virus disease (EVD) outbreak, and the tenth outbreak to occur in the Democratic Republic of the Congo (DRC), infected 3481 individuals and claimed 2290 lives. Of the infected individuals, 57% (n = 1970) were women.^1^ Because there is no evidence for a biologically driven difference in EVD risk by sex, the disproportionate number of women affected has been attributed to gendered norms and behaviors related to caregiving and decision-making roles within a household.^2^ There is, however, a paucity of gender-disaggregated data from which to examine EVD risk. Gender norms may influence exposure to EVD, intensity, and prognosis as well as personal protective behaviors against the virus. Risk reduction and control interventions need to account for mechanisms by which gender is associated with EVD transmission and outcomes.^2,3^

Individual self-protective and care-seeking behaviors can reduce the risk and transmission of EVD, including (1) preventive behaviors such as avoiding physical contact with those who are sick or dead, (2) agreeing to vaccination, and (3) seeking care when experiencing EVD-compatible symptoms. Risk-reductive behaviors can be influenced by a supportive environment that ensures access to EVD vaccination and high quality and safe medical care, EVD treatment centers, and accurate and timely information about the outbreak and containment measures.^4,5,6^

The mechanisms by which gender is associated with attitudes, perceptions, and preventive behaviors during epidemic outbreaks globally are under-researched. The present study aimed to characterize the role of self-reported gender identity in perceptions, attitudes, and behaviors regarding EVD during the 2018 to 2020 EVD outbreak in the eastern DRC, using detailed survey data collected from a high transmission region during the outbreak. We hypothesized that men and women differ in personal protective behaviors, including vaccine acceptance, health-seeking behaviors, and physical or social distancing, and that these differences are mediated by gender differences in levels of EVD information and knowledge, perceived disease risk, and social relations.

## Methods

### Study Design and Participants

This survey study was approved by the Massachusetts General Brigham Human Research Committee and ad hoc ethic committees at the Research Center on Democracy and Development in Africa, Free University of the Great Lakes Countries in the DRC. Oral rather than written consent was used to facilitate comprehension by participants, build trust, and reduce the risk of collecting identifiable information.

Study design and data collection have been described previously^7^ and are summarized here, following the American Association for Public Opinion Research (AAPOR) reporting guideline. Cross-sectional survey data were collected in EVD-affected areas in North Kivu province of eastern DRC from April 20, 2019, to May 10, 2019, a period of high EVD transmission. We used a multistage cluster random-sampling approach to identify participants. First, using comprehensive lists, we randomly selected 30 avenues or cells (clusters) per city, the lowest administrative unit. Then, 16 households with at base 1 adult participant (aged at least 18 years) per household were randomly selected per cluster. The sample size was calculated to estimate proportions on a few key perception and behavior indicators in the given population for a 95% CI and 10% precision. We used a 0.5 proportion estimate, and the sample size was multiplied by 2 for comparison by sex, as well as by the number of cities, and to account for an estimated design effect of 2. The sample size was increased by 20% to account for nonresponse and logistical constraints. Surveys were administered at the time of recruitment by a team of experienced university researchers trained in the conduct of interviews. If selected respondents were absent, 3 attempts were made to contact them over the course of the survey. To facilitate interviews, female and male interviewers were assigned to same sex respondents, providing equal representation in the sample. Participation was anonymous, voluntary, and without compensation. Interviewers were trained on preventive measures to reduce risk of exposure, and all households were provided printed information on transmission prevention and avoidance at the end of the questionnaire.

The standardized, structured questionnaire was developed in French, and translated into Swahili. Independent experts reviewed and validated the translation, and local experts established face validity. Pilot interviews were conducted to test and validate the questionnaire. Interviewers were experienced in community survey methods and participated in a 1-week training course on the questionnaire content and sampling protocol. Survey data were collected electronically using KoboToolbox (Harvard Humanitarian Initiative).

### Measures

The 3 main behavioral outcomes of interest were (1) EVD vaccine acceptance, (2) formal EVD health care seeking, and (3) EVD self-protective behaviors. A vaccine acceptance score was created by summing responses on 2 dichotomous survey items: belief that the vaccine was safe and acceptance of the vaccine, if offered. EVD health care–seeking was measured with an open-ended question that asked where, if at all, respondents would seek care if they suspected they had EVD. The 4 response categories were as follows: (1) formal EVD-equipped health services, including hospitals, health centers, and EVD treatment units; (2) formal but non-EVD-equipped services, including pharmacy or community health workers; (3) informal services, including traditional healers, religious leaders, friends, and family; and (4) no health care–seeking. EVD self-protection was computed as a sum score of key self-protective behaviors that participants reported engaging in since the outbreak began. These self-protective behaviors were represented by 16 dichotomous items relating to direct avoidance of EVD cases, targeted social distancing, general physical and social distancing, and hand hygiene. A complete listing of all behaviors is shown in Table 1.

**Table 1.** Sociodemographic Characteristics, EVD Information, Knowledge, Perceptions, and Behaviors (Item Responses for Mediator Variables)

	Unweighted No. (weighted %)			Pearson χ^2^	*P* value
	Total (n = 1395 [100%])	Women (n = 698 [50%])	Men (n = 697 [50%])		
Age group, y					
18-30	655 (47)	358 (50)	297 (44)	10.55	.005
31-45	456 (32)	309 (30)	247 (34)		
≥46	284 (21)	131 (19)	153 (22)		
Education					
None-incomplete primary	230 (17)	151 (21)	79 (12)	67.07	<.001
Primary completed	560 (40)	317 (46)	243 (34)		
Secondary completed	605 (43)	230 (33)	375 (54)		
Wealth					
None	302 (21)	198 (27)	104 (15)	65.65	<.001
Poorest	347 (25)	131 (19)	216 (31)		
Poor	333 (24)	138 (19)	195 (28)		
Rich	413 (30)	231 (35)	182 (26)		
Mobile phone, ownership of mobile phone	1114 (81)	515 (75)	599 (86)	32.05	<.001
Ethnicity, Nande	1286 (93)	643 (92)	643 (93)	0.009	.93
EVD information topics					
Cases of EVD in the province	1138 (84)	612 (91)	526 (77)	49.56	<.001
Response to EVD in the province	1139 (83)	610 (90)	529 (76)	44.74	<.001
Information about the symptoms of EVD	1129 (90)	615 (91)	614 (89)	1.32	.25
Information about how to prevent/protect yourself from EVD	1252 (92)	623 (92)	629 (92)	0.34	.56
Information about where to seek care for EVD	1199 (88)	612 (90)	587 (86)	9.10	.003
Information about what to do when someone close (family member, friend, neighbor) has contracted EVD	1160 (85)	593 (87)	567 (82)	7.88	.005
EVD symptoms					
High fever	1165 (85)	552 (82)	613 (89)	14.59	<.001
Acute headaches	940 (69)	443 (65)	497 (72)	6.93	.008
Muscular pain	449 (32)	355 (37)	194 (27)	14.14	<.001
Weakness	508 (37)	333 (48)	175 (25)	83.15	<.001
Tiredness	493 (35)	319 (46)	174 (24)	71.16	<.001
Diarrhea	1129 (83)	564 (83)	565 (82)	0.49	.49
Vomiting	1071 (79)	508 (75)	563 (82)	8.70	.003
Stomach/abdomen pain	219 (15)	91 (12)	128 (18)	6.63	.01
Bleeding	277 (21)	133 (21)	144 (21)	0.32	.57
EVD causes					
Virus	741 (54)	279 (41)	462 (67)	91.39	<.001
Eating/handling bush meat	608 (45)	296 (44)	312 (46)	0.31	.58
Witchcraft	114 (8)	97 (14)	17 (2)	62.94	<.001
Ancestral intervention	2 (0)	2 (0)	0	2.04	.15
God’s will	4 (0)	4 (1)	0	4.09	.04
EVD transmission					
Air	120 (9)	89 (14)	31 (4)	31.96	<.001
Eating/handling bush meat	885 (65)	414 (61)	471 (69)	7.8	.006
Physical contact with someone infected by EVD	1025 (74)	485 (71)	540 (77)	8.02	.005
Physical contact with someone who died of EVD	979 (70)	432 (62)	547 (79)	40.34	<.001
Contact with objects that came in contact with someone infected by EVD	811 (58)	372 (53)	439 (63)	10.68	.001
Contact with bodily fluids of someone infected by EVD	653 (47)	245 (35)	408 (58)	72.17	<.001
Sexual contact with someone infected by EVD	306 (22)	145 (20)	161 (23)	0.72	.40
Witchcraft	59 (5)	50 (8)	9 (1)	30.60	<.001
Ancestor intervention	0	0	0	NA	NA
God’s will	5 (0)	1 (0)	4 (1)	1.75	.19
Belief in EVD rumors					
Believe EVD does not exist	357 (27)	226 (34)	131 (20)	36.73	<.001
Believe EVD invented by authorities to have money	562 (43)	311 (48)	251 (38)	12.92	<.001
Believe EVD invented to destabilize region	549 (42)	296 (45)	253 (38)	7.09	.008
Quality of social relations (perceived as good or very good)					
Relationship with your parents, children, spouse	1305 (94)	666 (96)	639 (92)	8.07	.005
Relations with your neighbors	1158 (83)	598 (85)	560 (80)	7.02	.008
Relationships with people in your neighborhood, village	1100 (79)	597 (85)	503 (72)	37.35	<.001
Relationships with people in your ethnic group	1081 (77)	613 (88)	468 (66)	85.49	<.001
Relationships with members of any other ethnic group	845 (61)	450 (66)	395 (57)	8.88	.003
EVD risk perception					
Improbable disease threat (self, relative)	347 (24)	207 (28)	140 (20)	53.86	<.001
Undefined disease threat	664 (47)	284 (41)	380 (54)		
Probable other disease threat (with and without relative, without self)	196 (15)	130 (20)	66 (9)		
Probable disease threat (with and without relatives and others)	188 (14)	77 (11)	111 (17)		
EVD vaccine acceptance					
Believe it prevent or cures EVD	757 (53)	335 (48)	422 (59)	18.88	<.001
Accept vaccine	570 (40)	258 (37)	312 (43)	7.11	.008
EVD formal care-seeking					
None or undefined	180 (14)	142 (22)	38 (7)	106.52	<.001
Informal care (traditional healers or religious chiefs)	42 (3)	35 (5)	7 (1)		
Informal care (CHWs or pharmacy)	34 (2)	6 (1)	28 (4)		
Formal care (hospital or health center)	1109 (80)	493 (71)	616 (89)		
EVD self- protective behaviors					
Targeted physical distancing					
Avoid people you think may have recently visited an EVD-affected area	898 (66)	381 (57)	517 (75)	52.87	<.001
Avoid contact with people suspected to have EVD	1115 (82)	528 (78)	587 (85)	11.46	.001
Avoid contact with body of suspected EVD death	1144 (84)	526 (78)	618 (90)	35.52	<.001
Avoid contact with people suspected of recent contact with someone infected by EVD	896 (66)	375 (56)	521 (75)	61.38	<.001
General social and physical distancing					
Avoid visiting extended family members	71 (5)	54 (8)	17 (3)	21.09	<.001
Avoid visiting neighbors	87 (6)	76 (11)	11 (2)	53.20	<.001
Stay home more than usual	108 (8)	99 (14)	9 (1)	83.32	<.001
Keep children home from school	32 (2)	27 (4)	5 (1)	15.92	<.001
Reduce physical interactions with relatives	506 (35)	318 (46)	188 (24)	57.09	<.001
Reduce physical interactions with others	806 (59)	416 (62)	390 (56)	3.44	.06
Sexual avoidance (avoid/reduce sexual encounters, even with a spouse or partner)	184 (13)	101 (14)	83 (13)	2.45	.12
Avoid public spaces like markets or stadiums	167 (12)	151 (22)	16 (2)	127.30	<.001
Avoid going to church or mosque	91 (6)	61 (9)	30 (4)	11.96	.001
Avoid taking public transport	120 (9)	71 (10)	49 (7)	4.89	.03
Funeral (avoided attending a funeral)	382 (27)	206 (30)	176 (25)	4.11	.04
Hygiene (washing hands more frequently)	1026 (76)	544 (81)	482 (71)	20.22	<.001

Abbreviations: EVD, Ebola virus disease; NA, not applicable.

Our primary risk factor of interest was self-reported gender identity (men, women). Gender is used rather than biological sex to highlight the contextual factors related to gender norms and values that are associated with health behaviors. We also assessed the following sociodemographic factors: age group (18 to 30 years, 31 to 45 years, and at least 46 years), education (none, primary completed, secondary completed), wealth index (none, poorest, poor, and rich), ownership of mobile phone (yes, no), and ethnicity (Nande, other).

We constructed the following 5 potential mediators of the association between gender and the outcomes by combining the scores of individual items with similar themes: (1) EVD information awareness score, (2) EVD knowledge accuracy score, (3) EVD risk-perception score, (4) belief in rumors score, and (5) social relations score. The EVD information awareness score was quantified by the extent participants reported receiving information on EVD-related topics by summing responses on 6 binary (yes, no) items related to EVD prevention, symptoms, knowledge of where to seek health care, knowledge of actions to take if a relative or neighbor has EVD, updates on EVD in the province, and overall EVD response. The EVD knowledge accuracy score assessed knowledge of EVD symptoms, transmission modes, and causes. The score was computed as the total number of correct answers. The EVD risk-perception score measured perceived risk of contracting EVD within 30 days following survey administration by combining participant responses into 4 risk categories (improbable, undefined, probable to others, probable to self and relatives). The belief-in-rumors sum score combined binary items assessing whether participants believed that EVD was real, if it was invented by authorities for financial or other gains, or if it was invented to destabilize the region, which were commonly cited rumors in the DRC. The social relations score was a summary score comprising items on perceived quality of social relationships with family members, neighbors, and members of the same or other ethnic groups on a 5-point Likert scale from very bad to very good.

### Statistical Analysis

All analyses were conducted using the complex sample module in SPSS version 25 (IBM Corp) and survey data analyses in Stata version 16 (StataCorp) from August 2019 to May 2020. Data were weighted to reflect the unequal probability of sampling between cities, using the best available population estimates for the 3 cities. Frequencies, percentages, and statistically significant χ^2^ tests for gender differences are reported for categorical variables; weighted means, standard errors, and 95% CIs are reported for continuous variables (Table 1 and Table 2). We evaluated associations between mediator and outcome variables to assess collinearity (Table 3). Testing was 2-sided, and the threshold for statistical significance was *P* < .05.

**Table 2.** Mediator and Outcome Summary Scores

Variable (range of scores)	Weighted mean (SE) [95% CI]			Women vs men	
	Full sample	Women	Men	T-test	*P* value
EVD information awareness score (0-6)	5.22 (0.04) [5.13-5.30]	5.42 (0.06) [5.30-5.53]	5.02 (0.07) [4.89-5.15]	4.74	<.001
EVD knowledge accuracy score (1-20)	11.58 (0.10) [11.38-11.78]	11.08 (0.16) [10.77-11.39]	12.06 (0.13) [11.82-12.31]	−4.67	<.001
Sum of beliefs in EVD rumors (0-3)	1.12 (0.03) [1.05-1.18]	1.27 (0.05) [1.17-1.37]	0.97 (0.05) [0.87-1.06]	4.58	<.001
Quality of social relations (1-14)	10.61 (0.06) [10.50-10.73]	11.19 (0.09) [11.03-11.36]	10.04 (0.08) [9.88-10.19]	9.93	<.001
EVD risk perception (0-3)	1.19 (0.03) [1.14-1.24]	1.15 (0.04) [1.08-1.22]	1.23 (0.04) [1.15-1.30]	−2.01	.04
EVD vaccine acceptance (0-2)	0.94 (0.02) [0.89-0.98]	0.85 (0.04) [0.78-0.92]	1.02 (0.03) [0.95-1.09]	−3.91	.001
EVD formal care-seeking (0-3)	2.48 (0.03) [2.42-2.54]	2.22 (0.05) [2.12-2.31]	2.74 (0.03) [2.68-2.81]	−9.32	<.001
No. of EVD self-protective behaviors (0-15)	5.55 (0.08) [5.39-5.71]	5.78 (0.14) [5.50-6.06]	5.33 (0.09) [5.16-5.50]	2.73	.006

Abbreviation: EVD, Ebola virus disease.

**Table 3.** Correlation Between EVD Mediators and Outcomes

	EVD information awareness	EVD knowledge accuracy	Belief in EVD rumors	Quality of social relations	EVD vaccine acceptance	EVD risk perception	EVD formal care-seeking	EVD self-protective behaviors
EVD information awareness	1.00	NA	NA	NA	NA	NA	NA	NA
EVD knowledge accuracy	0.29	1.00	NA	NA	NA	NA	NA	NA
Belief in EVD rumors	−0.02	−0.39	1.00	NA	NA	NA	NA	NA
Quality of social relations	0.14	0.09	−0.21	1.00	NA	NA	NA	NA
EVD vaccine acceptance	0.10	0.14	−0.09	−0.15	1.00	NA	NA	NA
EVD risk perception	0.11	0.49	−0.46	0.18	0.12	1.00	NA	NA
EVD formal care-seeking	0.07	0.46	−0.29	0.11	0.08	0.42	1.00	NA
EVD self-protective behaviors	0.33	0.41	−0.16	0.09	0.06	0.21	0.24	1.00

Abbreviations: EVD, Ebola virus disease; NA, not applicable.

We conducted path analyses using a structural equation modeling framework to examine associations among variables. We used the full information maximum likelihood (FIML) estimator to account for item-specific nonreponse (refusal) found in 70 (5.0%) of the questionnaires; survey weights to account for sampling probability; and robust standard errors to deal with non-normality of the data after the null hypothesis for multivariate normality was rejected using the Doornik-Hansen test. We calculated goodness-of-fit indices to assess model fit: root mean square error of approximation (RMSEA) (best if less than 0.06), comparative fit index (CFI) (best if greater than 0.90), and the coefficient of determination (CD) (best if greater than 0.08).^8^ Variables of interest were selected based on theoretical considerations and previous studies.^2,3,7,9,10^ All pathways between gender, mediators, and outcomes were included in the final model, and pathways between demographic covariates and mediators were finalized based on exploratory stepwise regression and modification indices.

We examined associations (eFigure in the Supplement) between the key risk factor (gender), mediators (EVD information awareness, EVD knowledge accuracy, EVD risk-perception, belief in rumors, and social relations), and EVD outcomes (vaccine acceptance, formal care-seeking, and self-protective behaviors). We assessed the association of gender with each variable, along with associations of all risk factors and mediators with each outcome. We tested for mediation associations by examining indirect pathways between gender and each outcome through the mediator variables, including location, education, and wealth. Covariances were freely estimated among all risk factors, as well as between error terms.

## Results

A total of 1419 households were approached, among which 8 (0.6%) had no available participants and 16 (1.1%) refused participation. Individual participants were approached in 1395 households (98.3%). Among the selected households, a total of 1420 individuals were approached, among who 6 were not available after multiple attempts, and 19 refused to participate.

The final sample included 1395 participants, all with completed interviews, residing in the cities of Beni, Butembo, and Katwa in eastern DRC. Participants’ mean (SD) age was 34.5 (13.1) years. For the sociodemographic risk factors, men reported higher levels of educational attainment and mobile phone ownership (Table 1). We found significant differences in topics of EVD-related information received by men and women (Table 1). More women than men reported receiving EVD information about cases in the province (612 women [91%] vs 526 men [77%]; χ^2^ = 49.56; *P* < .001), response efforts (610 women [90%] vs 529 men [76%]; χ^2^ = 44.74; *P* < .001), where to seek care (612 women [90%] vs 587 men [86%]; χ^2^ = 9.10; *P* = .003), and dealing with EVD cases (593 women [87%] vs 567 men [82%]; χ^2^ = 7.88; *P* = .005). Women were more likely than men to believe EVD rumors (eg, 226 women [34%] believed that EVD does not exist vs 131 men [20%]; χ^2^ = 36.73; *P* < .001).

More men than women thought that vaccines could prevent and cure EVD (422 men [59%] vs 335 women [48%]; χ^2^ = 18.88; *P* < .001) and were more willing to accept the vaccine (312 women [43%] vs 258 men [37%]; χ^2^ = 7.11; *P* = .008) (Table 1). Men were more likely to seek formal health care for EVD (616 men [89%] vs 493 women [71%]; χ^2^ = 106.52; *P* < .001). Men reported engaging in more direct avoidance behaviors for EVD self-protection (eg, avoid contact with people suspected of recent contact with someone infected by EVD: 521 men [75%] vs 375 women [56%]; χ^2^ = 61.38; *P* < .001), whereas women reported engaging in more general physical and social distancing such as physical interaction avoidance (eg, reduce physical interactions with relatives: 318 women [46%] vs 188 men [24%]; χ^2^ = 57.09; *P* < .001), public space avoidance (151 women [22%] vs 16 men [2%]; χ^2^ = 127.30; *P* < .001), and funeral avoidance (206 women [30%] vs 176 men [25%]; χ^2^ = 4.11; *P* = .04). Women reported more handwashing compared with men (544 women [81%] vs 482 men [71%]; χ^2^ = 20.22; *P* < .001).

We found women, compared with men, had higher mean (SE) scores for reported EVD information awareness (women: 5.42 [0.06] vs men: 5.02 [0.07]; *P* < .001), belief in rumors (women: 1.27 [0.05] vs men: 0.97 [0.05]; *P* < .001), quality of social relations (women: 11.19 [0.09] vs men: 10.04 [0.08]; *P* < .001), and self-protective behaviors (women: 5.78 [0.14] vs men: 5.33 [0.09]; *P* = .006) in the overall sample (Table 2). Men, compared with women, displayed higher EVD knowledge accuracy (men: 12.06 [0.13] vs women: 11.08 [0.16]; *P* < .001), risk perception (men: 1.23 [0.04] vs women: 1.15 [0.13]; *P* = .04), EVD vaccine acceptance (men: 1.02 [0.03] vs women: 0.85 [0.04]; *P* = .001), and formal health care seeking (men: 2.74 [0.03] vs women: 2.22 [0.05]; *P* < .001).

### Path Analysis

The estimated direct and indirect associations from the path analyses are presented in Table 4, and pathways are depicted in the Figure. Model fit statistics indicated adequate fit for the path analysis model (RMSEA = 0.08; CFI = 0.92; CD = 0.26) and additional fit indices are reported in the eTable in the Supplement.

**Table 4.** Path Analysis Coefficients for Direct and Indirect Associations

	EVD vaccine acceptance		EVD formal care-seeking		EVD self-protection behaviors	
	β	*P* value	β	*P* value	β	*P* value
EVD information awareness	−0.03	.26	−0.02	.39	0.23	<.001
EVD knowledge accuracy	0.37	<.001	0.39	<.001	0.35	<.001
Belief in EVD rumors	−0.3	<.001	−0.09	.007	−0.04	.21
Quality of social relations	0.11	<.001	0.09	<.001	−0.01	.7
EVD risk perception	0.05	.04	0.02	.54	−0.004	.83
Gender						
Direct effect	0.03	.3	0.2	<.001	−0.1	<.001
Indirect effect	0.05	.005	0.03	.04	0.01	.58

Abbreviations: β, standardized beta regression coefficient; EVD, Ebola virus disease.

**Figure.**
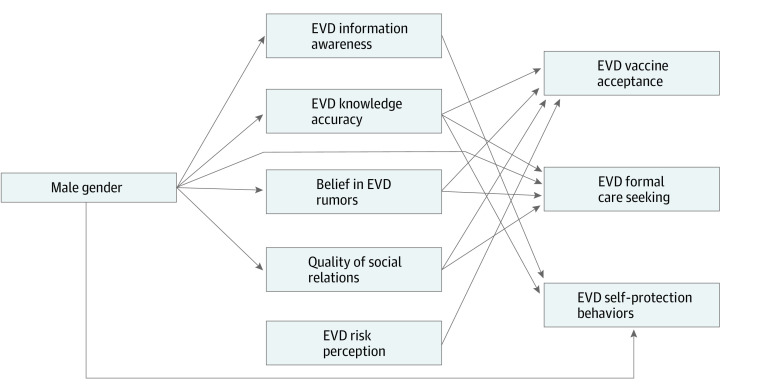
Pathways Between Gender, Mediators, and Outcomes in the High-EVD Cities (Beni, Butembo, Katwa; N = 1395) in May 2019 Other factors (education, wealth, mobile) were included in the model, but not shown in this figure. Only statistically significant (*P* < .05) pathways are shown in this figure. β coefficients for the pathways are in Table 4. EVD indicates Ebola virus disease.

#### Mediator-Outcome Direct Associations

Higher EVD knowledge accuracy was associated with increases in all 3 EVD outcomes: vaccine acceptance (β = 0.37; *P* < .001), formal care seeking (β = 0.39; *P* < .001), and self-protective behaviors (β = 0.35; *P* < .001). Lower believe in EVD rumors was associated with higher EVD vaccine acceptance (β = −0.30; *P* < .001) and greater formal care seeking for EVD (β = −0.09; *P* = .007); and better social relations was also associated with higher EVD vaccine acceptance (β = 0.11; *P* < .001) and greater formal care seeking for EVD (β = 0.09; *P* < .001). Engagement in EVD self-protective behaviors was also associated with higher EVD information awareness (β = 0.23; *P* < .001), and vaccine acceptance was also associated with higher EVD risk perception (β = 0.05; *P* = .04).

#### Gender Direct Associations

Women reported engaging in more self-protective behaviors. Women also reported higher EVD information awareness, greater belief in EVD rumors, and better social relationships. Men reported more formal care-seeking and registered higher EVD knowledge scores.

#### Gender Indirect Associations

We identified an indirect association for men having higher EVD vaccine acceptance and seeking more formal modes of EVD care than women, with mediation for both outcomes through higher EVD knowledge accuracy, lower belief in EVD rumors, and despite lower quality social relations.

## Discussion

The tenth and largest EVD epidemic in the DRC had a devastating impact on a region experiencing protracted armed conflict and political crises for more than a decade. This study examines the pathways by which gender differences in EVD-related information, knowledge, and perceptions are associated with risk-related attitudes and behaviors. We analyzed data collected during the EVD outbreak with the aim of better understanding social and behavioral factors associated with EVD-related gender disparities.

Women reported higher EVD information awareness but also greater belief in EVD rumors than men. This is consistent with previous findings from Ebola-affected areas conducted during the beginning of the outbreak.^7^ Conversely, men reported more accurate knowledge of EVD symptoms, causes, and transmission compared with women. Unpublished qualitative interviews found that most of the first waves of EVD-related training and communication implemented by the government and responding organizations were targeted at men due to their greater access to formal communication channels.

Greater EVD knowledge accuracy was, in turn, associated with increases in all 3 EVD preventive behavioral outcomes: vaccine acceptance, formal care seeking, and self-protective behaviors. Lower belief in EVD rumors was associated with greater vaccine acceptance, and greater EVD information awareness was associated with increased adoption of self-protective behaviors. These findings reinforced the important role of accurate and targeted information in outbreak control and prevention.^7,11,12,13^

We found associations between gender and the EVD preventive behavioral outcomes, as well as evidence for mediation of the gender associations through the pathways of EVD knowledge and belief in rumors. Men reported overall higher EVD vaccine acceptance and formal EVD health care seeking, which was mediated by their higher reported EVD knowledge accuracy and lower belief in rumors (the strongest factors associated with vaccine acceptance) compared with women. Nevertheless, women reported engaging in a greater number of self-protective behaviors compared with men.

These gender differences reinforce previous findings on the role of greater trust and reduced misinformation in vaccine acceptance.^14,15^ The findings suggest that increasing vaccine acceptance requires engaging in transparent and targeted communication to support individuals in making informed decisions rather than pressuring people to receive vaccines. Women may have been less informed about vaccines because communication materials about vaccines were not available in the appropriate format. Early decisions to exclude pregnant and breast-feeding women from accessing vaccines, with limited communication, and ultimately a policy reversal created misunderstanding and suspicion among women.^16,17^

There are several potential barriers to care-seeking in this population. Free health care appears to have encouraged care-seeking.^18^ Women, however, may have been more likely than men to avoid formal health care facilities.^19^ We found that greater perceived EVD risk and higher EVD knowledge accuracy were the most robust factors associated with formal health care seeking for EVD symptoms. This supports qualitative research from the West Africa EVD outbreaks that found fear of the disease and misinformation as the biggest barriers to treatment seeking.^6,20^ Higher EVD knowledge accuracy and greater acceptance of formal health care services among men, compared with women, is consistent with research describing access to accurate EVD information for women.^21^ Mobility for treatment-seeking among women may further be reduced in areas of armed conflict and heightened insecurity, due to gender-based violence and attacks on health facilities and health care workers.^22^

Greater EVD information and knowledge was found to be associated with the adoption of more self-protective behaviors. Cultural and social norms indicate that women are more likely to be caregivers, prepare food, and have greater contact with dead bodies during traditional burial practices.^2,3,23^ This may explain why women engaged more frequently than men in self-protective behaviors.

Calls to integrate women and gender-related concerns into the response to disease outbreaks have emerged,^9,24,25^ echoing broader issues on the provision of care, accessibility, and reporting of gender-disaggregated data^9,24^ and highlighting the need to address male bias in policy and planning.^9^ The response in DRC, apart from community dialogues for women, did not sufficiently target nor engage women, nor did it adapt the medium of its messaging to facilitate improved communication with women. In future responses to infectious disease outbreaks such as COVID-19, it is critical to engage women in the response and communication strategy.

### Limitations

This study has some limitations. First, the study is limited to urban areas in eastern DRC and may not be generalizable to rural or culturally distinct areas. Although our analyses adjusted for key factors that may be associated with gender, social, and behavioral EVD outcomes, the possibility of unmeasured confounders cannot be ruled out. These may include women’s caregiving roles, gender norms, mobility and access to care, and involvement in funeral practices, among others. Additionally, the data were self-reported and may be subject to social desirability, information, and selection biases, which should be taken into consideration when interpreting the results. Interviews were conducted in Swahili and/or French, which are the commonly spoken languages in the urban area under study, but not necessarily the preferred language of respondents. The instrument and training of interviewers were designed to reduce risks of bias. Since the variables under study relate to perceptions and attitudes, self-reporting was considered an appropriate data collection method.

## Conclusions

The study data were collected at the peak of the EVD outbreak in DRC using a rigorous multistage cluster random-sampling process. The quantitative analysis used a structural equation modeling framework because of its ability to simultaneously assess multiple direct and indirect associations for a range of variables, to compare statistical fit across models, and to establish and test a comprehensive risk pathway framework for health behaviors.

The results provide insights into the sociocultural dimensions of gender vulnerabilities and EVD risk, while also examining the mediating role of EVD-related information, knowledge, and perceptions in the association between gender and EVD preventive behaviors. Self-protective behaviors including vaccine acceptance are the cornerstone of EVD control, especially given limited therapeutic options. Understanding gender-related factors associated with protective behaviors can help to shape outbreak control interventions. Our findings contribute to a better understanding of the role of gender in EVD control interventions.

## Notes

Supplement. **eFigure.** Conceptual Model **eTable.** Additional Model Fit Statistics

## References

[1] World Health Organization. Ebola virus disease – Democratic Republic of the Congo. Accessed January 14, 2022. https://www.who.int/emergencies/disease-outbreak-news/item/2021-DON351

[2] WHO. Addressing Sex and Gender in Epidemic-Prone Infectious Diseases. World Health Organization; 2007.

[3] Nkangu MN, Olatunde OA, Yaya S. The perspective of gender on the Ebola virus using a risk management and population health framework: a scoping review. Infect Dis Poverty. 2017;6(1):135. doi:10.1186/s40249-017-0346-7 29017587 PMC5635524

[4] Verelst F, Willem L, Beutels P. Behavioural change models for infectious disease transmission: a systematic review (2010-2015). J R Soc Interface. 2016;13(125):20160820. doi:10.1098/rsif.2016.0820 28003528 PMC5221530

[5] Malvy D, McElroy AK, de Clerck H, Günther S, van Griensven J. Ebola virus disease. Lancet. 2019;393(10174):936-948. doi:10.1016/S0140-6736(18)33132-5 30777297

[6] Carter SE, O’Reilly M, Walden V, Frith-Powell J, Umar Kargbo A, Niederberger E. Barriers and enablers to treatment-seeking behavior and causes of high-risk practices in Ebola: a case study from Sierra Leone. J Health Commun. 2017;22(sup1):31-38. doi:10.1080/10810730.2016.1222034 28854134

[7] Vinck P, Pham PN, Bindu KK, Bedford J, Nilles EJ. Institutional trust and misinformation in the response to the 2018-19 Ebola outbreak in North Kivu, DR Congo: a population-based survey. Lancet Infect Dis. 2019;19(5):529-536. doi:10.1016/S1473-3099(19)30063-5 30928435

[8] Hu L, Bentler PM. Cutoff criteria for fit indexes in covariance structure analysis: conventional criteria versus new alternatives. Structural Equation Modeling: A Multidisciplinary Journal. 1999. 6:1-55. doi:10.1080/10705519909540118

[9] Harman S. Ebola, gender and conspicuously invisible women in global health governance. Third World Q. 2016;37(3):524-541. doi:10.1080/01436597.2015.1108827

[10] Kankya C, Nabadda D, Kabonesa C, . Social dynamics of Ebola virus disease: a case of Bundibugyo District, Uganda. Health (N Y). 2019;11(1):108-128. doi:10.4236/health.2019.111011

[11] Tenkorang EY. Effect of knowledge and perceptions of risks on Ebola-preventive behaviours in Ghana. Int Health. 2018;10(3):202-210. doi:10.1093/inthealth/ihy009 29506203

[12] Oppenheim B, Lidow N, Ayscue P, . Knowledge and beliefs about Ebola virus in a conflict-affected area: early evidence from the North Kivu outbreak. J Glob Health. 2019;9(2):020311. doi:10.7189/jogh.09.020311 31656600 PMC6812978

[13] Jalloh MF, Sengeh P, Monasch R, . National survey of Ebola-related knowledge, attitudes and practices before the outbreak peak in Sierra Leone: August 2014. BMJ Glob Health. 2017;2(4):e000285. doi:10.1136/bmjgh-2017-000285 29259820 PMC5728302

[14] Irwin KL, Jalloh MF, Corker J, ; 2015 Guinean Household Survey of Ebola Virus Disease Project Group. Attitudes about vaccines to prevent Ebola virus disease in Guinea at the end of a large Ebola epidemic: results of a national household survey. Vaccine. 2017;35(49 Pt B):6915-6923. doi:10.1016/j.vaccine.2017.06.026 28716555

[15] Larson HJ, Jarrett C, Eckersberger E, Smith DMD, Paterson P. Understanding vaccine hesitancy around vaccines and vaccination from a global perspective: a systematic review of published literature, 2007-2012. Vaccine. 2014;32(19):2150-2159. doi:10.1016/j.vaccine.2014.01.081 24598724

[16] Haddad LB, Jamieson DJ, Rasmussen SA. Pregnant women and the Ebola crisis. N Engl J Med. 2018;379(26):2492-2493. doi:10.1056/NEJMp1814020 30485156

[17] Bebell LM, Oduyebo T, Riley LE. Ebola virus disease and pregnancy: a review of the current knowledge of Ebola virus pathogenesis, maternal, and neonatal outcomes. Birth Defects Res. 2017;109(5):353-362. doi:10.1002/bdra.23558 28398679 PMC5407292

[18] Hung YW, Law MR, Cheng L, . Impact of a free care policy on the utilisation of health services during an Ebola outbreak in the Democratic Republic of Congo: an interrupted time-series analysis. BMJ Glob Health. 2020;5(7):e002119. doi:10.1136/bmjgh-2019-002119 32718948 PMC7389747

[19] McKay G , Black B, Mbambu Kahamba S, Wheeler E, Mearns S, Janvrin A. Not all that bleeds is Ebola: how has the DRC Ebola outbreak impacted sexual and reproductive health in North-Kivu? Accessed November 11, 2020. https://www.rescue.org/sites/default/files/document/4416/srhebolareport1172020.pdf

[20] UNICEF. Social science support for COVID-19 - Lessons Learned Brief 4 - social sciences evidence on barriers to.pdf. Accessed February 2, 2021. https://reliefweb.int/sites/reliefweb.int/files/resources/Social%20science%20support%20for%20COVID-19%20-%20Lessons%20Learned%20Brief%204%20-%20Social%20sciences%20evidence%20on%20barriers%20to.pdf

[21] Manivannan A. Gender inequalities in access to information about Ebola as gender-based violence. Harv Hum Rights J. Published June 2015. Accessed April 17, 2020. https://harvardhrj.com/2015/06/gender-inequalities-in-access-to-information-about-ebola-as-gender-based-violence/

[22] O’Brien M, Tolosa MX. The effect of the 2014 West Africa Ebola virus disease epidemic on multi-level violence against women. Int J Hum Rights Healthc. 2016;9(3):151-160. doi:10.1108/IJHRH-09-2015-0027

[23] Hewlett BS, Amola RP. Cultural contexts of Ebola in northern Uganda. Emerg Infect Dis. 2003;9(10):1242-1248. doi:10.3201/eid0910.020493 14609458 PMC3033100

[24] Davies SE, Bennett B. A gendered human rights analysis of Ebola and Zika: locating gender in global health emergencies. Int Aff. 2016;92(5):1041-1060. doi:10.1111/1468-2346.12704

[25] Smith J. Overcoming the ‘tyranny of the urgent’: integrating gender into disease outbreak preparedness and response. Gend Dev. 2019;27(2):355-369. doi:10.1080/13552074.2019.1615288

