# Supplementary material for: Protective Behaviors Associated With Gender During the 2018-2020 Ebola Outbreak in Eastern Democratic Republic of the Congo
Source: JAMA Netw Open. 2022 Feb 16;5(2):e2147462. doi: 10.1001/jamanetworkopen.2021.47462 (PMC8851299; doi:10.1001/jamanetworkopen.2021.47462)
Supplement: Supplement. — eFigure. Conceptual Model eTable. Additional Model Fit Statistics [file jamanetwopen-e2147462-s001.pdf]

Supplemental Online Content

Pham PN, Sharma M, Bindu KK, et al. Protective behaviors associated with gender during the 2018-2020 Ebola outbreak in Eastern Democratic Republic of the Congo. *JAMA Netw Open*. 2022;5(2):e2147462. doi:10.1001/jamanetworkopen.2021.47462

eFigure. Conceptual Model
eTable. Additional Model Fit Statistics

This supplemental material has been provided by the authors to give readers additional information about their work.

© 2022 Pham PN et al. *JAMA Network Open.*

eFigure. Conceptual Model

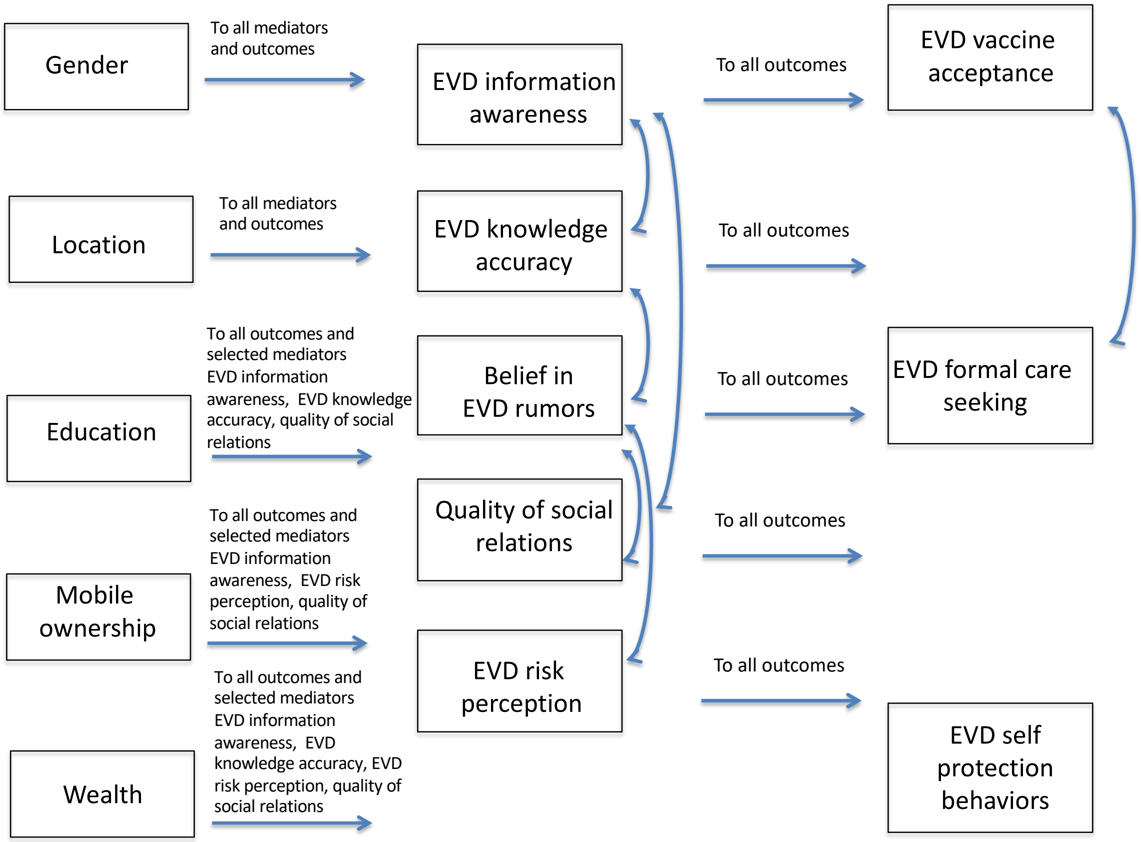

Abbreviation: EVD, Ebola Virus Disease

© 2022 Pham PN et al. *JAMA Network Open.*

eTable: Additional Model fit statistics

	High EVD Risk (N=1395)
**RMSEA**	0.08
**CFI**	0.92
**TLI**	0.75
**CD**	0.26
**AIC**	49635.94
**BIC**	49929.41

Abbreviations: RMSEA, Root mean square error of approximation; CFI, Comparative fit index; TLI,Tucker lewis index; CD, coefficient of determination; AIC, Akaike information criteria; BIC, Bayesian information criteria

© 2022 Pham PN et al. *JAMA Network Open.*
